# Cross-Sectional Study on Lateral Skull Radiographs to Design a New Nasopharyngeal Swab for Simplified COVID-19 and Respiratory Infections Diagnostic Testing in Children

**DOI:** 10.3390/jcm12010213

**Published:** 2022-12-27

**Authors:** Cecilia Goracci, Alessandra Volpe, Lorenzo Salerni, Elisabetta Paolini, Alessandro Vichi, Lorenzo Franchi

**Affiliations:** 1 Department of Medical Biotechnologies, University of Siena, 53100 Siena, Italy; 2 Department of Medical, Surgical, and Neurological Sciences, University of Siena, 53100 Siena, Italy; 3 Department of Experimental and Clinical Medicine, The University of Florence, 50127 Florence, Italy; 4 Dental Academy, University of Portsmouth, Portsmouth O1 2QG, UK

**Keywords:** nasopharyngeal swab, sample collection, COVID-19, SARS-CoV-2, respiratory infections, children, lateral skull radiograph

## Abstract

Nasopharyngeal swab sample collection is the first-line testing method for diagnosing COVID-19 infection and other respiratory infections. Current information on how to properly perform nasopharyngeal swabbing in children is largely defective. This study aimed at collecting nostril to nasopharynx distance measurements on lateral skull radiographs of children and adolescents to design a nasopharyngeal swab meant to standardize and facilitate the sample collection procedure. A total of 323 cephalograms of 152 male and 171 female children aged 4–14 years taken for orthodontic reasons were selected. On each cephalogram, the shortest distance between the most anterosuperior point of the nostril contour and the nasopharynx outline was measured in mm parallel to the palatal plane. Descriptive statistics of the measurements were calculated for each age group. The lower limit of the 95% confidence intervals of the measurements was taken as a reference to design a swab shaft with marks that, at each age, delimitate a safety boundary for swab progression up to the posterior nasopharyngeal wall. The simplification of the procedure enabled by the newly designed nasopharyngeal swab is valuable to help healthcare providers perform specimen collection on children in a safe and effective way, perhaps under the less-than-ideal conditions possibly occurring in ‘point-of-need’ contexts.

## 1. Introduction

Currently, the global strategy to contain the spread of the COVID-19 pandemic is based on the early detection of suspected cases, early diagnosis of symptomatic patients, and the isolation of infected patients. For the confirmation of COVID-19 infection cases, the World Health Organization recommends performing nucleic acid amplification tests (NAAT test) with the reverse polymerase chain reaction technique (RT-PCR) on respiratory secretion samples collected with nasopharyngeal or oropharyngeal swab [1]. However, a nasopharyngeal swab has been indicated as the first-choice method of collection for the sensitivity of the diagnostic test [2,3,4].

Nasopharyngeal swab has been routinely used in the past for the diagnosis of viral upper respiratory tract infections in adults and children and has generally been performed in hospitalized symptomatic patients by trained personnel. However, the need for large-scale diagnostic tests, imposed by the pandemic spread of the COVID-19 infection, has required the rapid training of health personnel assigned to perform sample collection not only in hospital or ‘point-of-care’ settings but also, for screening purposes, in ‘point-of-need’ contexts, such as schools, ports, airports, and parking lots [2,5].

It has been reported that the training of the sample collection personnel was sometimes hasty and limited to just viewing educational videos [3,6,7,8,9,10,11,12]. However, proper sample collection is the most important step in the laboratory diagnosis of infectious diseases. An incorrectly collected sample can lead to false negative results, which represent a serious risk for containing the epidemic [5,13,14]. The information available in the current literature on the correct method to perform nasopharyngeal swab collection is rather general [5,6,15,16,17], especially concerning the depth of penetration of the swab, which should reach the mucosa of the posterior wall of the nasopharynx [5,15,16]. It has been stated that the depth of insertion of the swab along the nasal cavity floor should be about the same as the distance between the nostril and the ear [6,18]. External acoustic meatus, lobe, or tragus was taken as the ear landmark in different studies [6,17,18]. As for the nose landmark, the reference was generically made to the nostril or the philtrum [18,19]. However, in the study by Lim et al. [19], it was reported that the correlation between philtrum-tragus distance and nostril-nasopharynx distance is low and that between these two measures, there is a difference of about 5 cm in both genders. The same authors, by means of a nasal endoscope inserted in the inferior meatus, measured an average distance of 9.4 and 10 cm between the nostril and the posterior upper nasopharyngeal mucosa in adult female and male subjects, respectively [19]. Callesen et al., based on endoscopic measurements, reported that in a sample of adults, the mean insertion depth for nasopharyngeal swabbing was 9.4 cm [20]. According to Pondaven-Letourmy et al., in adults, the distance between the nostril and the posterior wall of the nasopharynx is between 8 and 10 cm, while for children, the same authors report the nostril to nasopharynx distance to be 6–7 cm [5]. However, such distance can be expected to change with age in relation to maxillary growth. It would, therefore, be useful to collect statistical data on the nostril to nasopharynx distance at different age intervals, with the aim of providing the healthcare professionals who are to test children and adolescents with useful guidelines to perform the swab progression inside the nasal cavity in an accurate, safe, and repeatable way.

Based on the collected data, a swab shaft can be designed with marks that signpost the advisable depth of insertion of the swab within the inferior nasal meatus at the different ages of childhood and adolescence to more predictably reach the posterior nasopharynx for proper sampling, without any trauma or excessive discomfort for the young patient.

## 2. Materials and Methods

The research was a retrospective cross-sectional study approved by the Pediatric Ethics Committee of Tuscany, Italy (No. 81/2021 of 23/03/2021).

**Figure 1:**
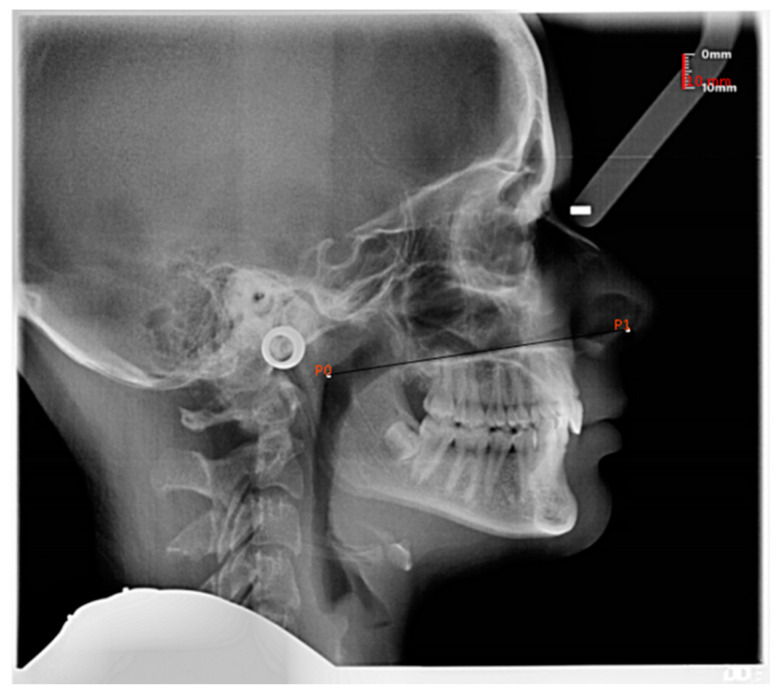
Measurement of the nostril to nasopharynx distance in a subject without adenoid hypertrophy. The measurement was recorded in mm from the most anterosuperior point of the nostril contour (P1) to the nasopharynx outline (P0), parallel to the palatal plane.

The study sample included 323 lateral skull radiographs taken for orthodontic purposes in 152 male and 171 female children of Caucasian race, aged between 4 years and 2 months and 14 years. The radiographs were derived from the database of the orthodontic private practice of one of the authors (CG), as well as from the Orthodontic Clinic of the University Hospital of Careggi in Florence. The subjects’ parents had given their informed consent to the use of the radiographs for the study. Subjects with severe systemic diseases and craniofacial deformities were excluded from the sample. Different skeletal or dental malocclusions, breathing patterns, habits, adenoid hypertrophy, enlarged tonsils, or snoring were not considered exclusion criteria. All the radiographs had been taken with the use of a cephalostat, and the subject was positioned with the Frankfort horizontal plane parallel to the floor and the teeth in centric occlusion. Selected cephalograms were of good quality, with the airway clearly visible and the subject is not swallowing. Measurements were calibrated by means of the ‘Calibration’ tool of the DeltaDent 2.2.0 software (Outside Format, Spino d’Adda, CR, Italy). The ‘Free measurement’ tool of the same software was utilized to measure, in mm, the shortest distance between the most anterosuperior point of the nostril contour and the nasopharynx outline parallel to the palatal plane (line connecting the anterior nasal spine and posterior nasal spine) (Figure 1).

**Figure 2:**
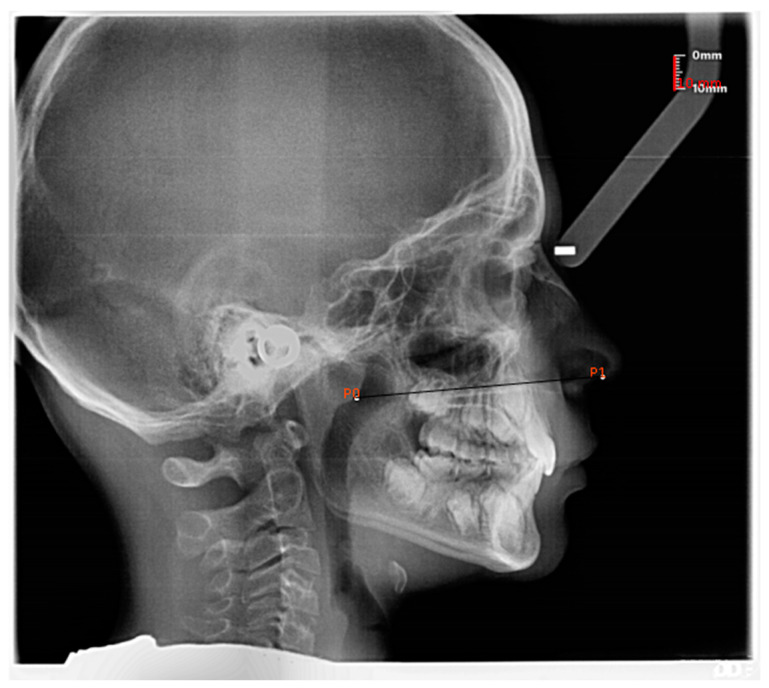
Measurement of the nostril to nasopharynx distance in a subject with adenoid hypertrophy. In the presence of adenoid hypertrophy, the posterior landmark was taken on the outline of the adenoid tissue.

In subjects exhibiting adenoid hypertrophy, the posterior landmark was taken on the anterior outline of adenoid tissue [21,22] (Figure 2).

One investigator (AVo) made all the measurements. Forty randomly selected cephalograms were traced a second time two weeks apart, 20 by the same investigator and 20 by a different investigator (EP), in order to determine intra- and inter-observer reproducibility through the calculation of intra-class correlation coefficients (ICCs).

Descriptive statistics of the nostril to nasopharynx distance data were calculated for each age group separately, and within each age group, the data were stratified by gender and presence of adenoid hypertrophy (Table 1). The statistical significance of the linear correlation between nostril to nasopharynx distance and age was verified with Pearson’s correlation coefficient test. Having verified that data distribution was normal according to the Kolmogorov–Smirnov test, the *t*-test for independent samples was applied within each age group to verify whether a statistically significant difference existed in the measured distance between males and females. Only in the age-4 group, as the data distribution was found to be non-normal, was the Mann-Whitney U test applied for between-gender comparison. In all the analyses, the level of significance was set at *p* < 0.05. Statistical calculations were handled by the PASW Statistics 18.0.0. software (SPSS Inc., Chicago, IL, USA).

## 3. Results

The ICCs were 0.99 and 0.98 for intra- and inter-examiner repeatability, respectively.

**Figure 3:**
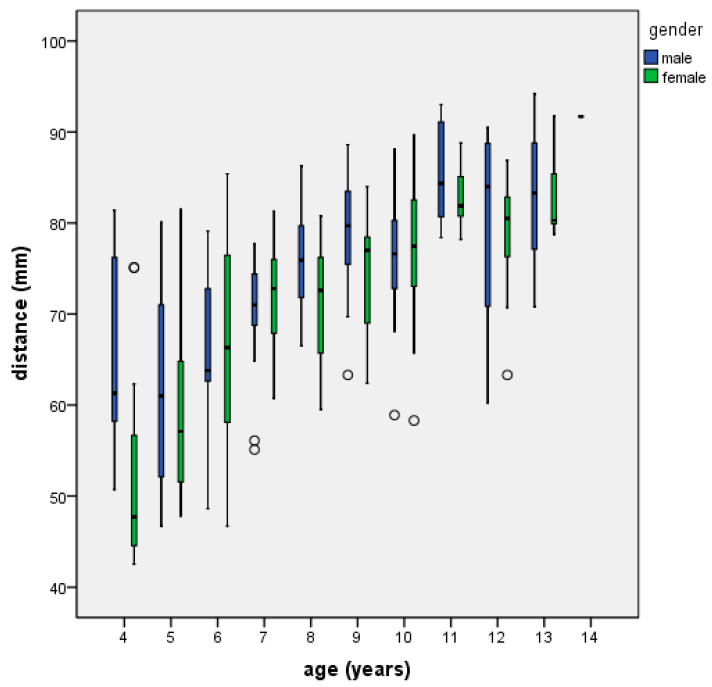
Boxplot displaying the descriptive statistics of the nostril to nasopharynx distance (mm) according to age (years) and gender.

**Figure 4:**
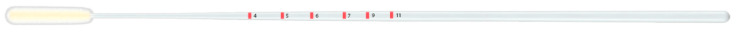
The newly designed nasopharyngeal swab, featuring marks along the shaft to signpost the proper depth of penetration along the nasal floor at different ages. The mark next to the number corresponding to the patient’s age should be aligned with the nostril.

Table 1 reports the descriptive statistics (mean, standard deviation, and 95% confidence interval) of the nostril to nasopharynx distance data by age group, gender, and adenoid hypertrophy. Figure 3 graphically displays the descriptive statistics of the nostril to nasopharynx distance by age and gender. A positive correlation existed between nostril to nasopharynx distance and age (Pearson’s Correlation Coefficient *p* = 0.644). The correlation was statistically significant (*p* < 0.001). No statistically significant difference emerged between males and females in any age range except 8–10 years. In this age interval, males had a longer nostril to nasopharynx distance than females. The occurrence of adenoid hypertrophy was more common between 7 and 9 years of age. Based on the 95% confidence intervals of the nostril to nasopharynx distance data collected for the different age groups, a swab shaft was designed with marks indicating the proper depth of penetration at different ages of childhood and adolescence (Figure 4). Specifically, the mark is located at a distance from the shaft tip equal to the lower bound of the 95% confidence interval of the nostril to nasopharynx distance at that age (Figure 4). Thereby, when the specific age mark is aligned with the nostril’s contour, the operator is 95% confident that the swab tip is reaching the nasopharynx or is slightly short of it. Thus, the mark provides a safety limit, guiding the further advancement of the swab up to reach the posterior nasopharyngeal wall in a harmless way.

The marks are painted with colored ink along the molded plastic shaft that ends with a tip coated with nylon fibers (Figure 4) [23].

The authors CG, AVi, and LF are the inventors of several embodiments of the aforementioned swab shaft, currently patent pending.

## 4. Discussion

Nasopharyngeal swab testing is currently the most commonly performed and most largely validated diagnostic method for COVID-19 infection [5,9]. An accurate nasopharyngeal collection procedure is, therefore, paramount for its substantial clinical and epidemiological implications.

Inadequate sampling can yield false negative results, which bring about a relevant risk of disease spread. Particularly, it is crucial to properly collect upper airway secretions by reaching the nasopharyngeal region since the expression of ACE2 receptors is higher in this area than in the proximal part of the nose [17,24]. Proper education and training are needed to enable the sample collection teams to reliably and safely perform sample collection in the proper section of the nasopharynx [18]. With this objective, any effort toward standardization and control of the sampling procedure is valuable, especially when dealing with children, whose reduced compliance with the unpleasant maneuver of swab penetration may further burden the technique sensitivity of the test [25]. Effective sampling is also particularly critical with children due to the purportedly high and still probably underestimated rate of asymptomatic carriers.

It can additionally be predicted that the need for COVID-19 diagnostic testing of children will remain in the near future, although vaccination campaigns are ongoing in several countries, as the pediatric age will be the last age category to be targeted by this preventive measure.

Furthermore, COVID-19 large-scale testing of children will still long be requested for their admittance to school, sport, and hobby activities, as well as to travel across countries. Such screening procedures will oftentimes be performed in ‘point-of-need’ contexts, perhaps under less-than-ideal sample collection conditions.

Even after the COVID-19 pandemic emergency is overcome, healthcare providers will still resort to nasopharyngeal swabbing as the appropriate test for differential diagnosis of several respiratory infections, which are particularly common in the pediatric age.

The current study stemmed from the discernment that the information available to healthcare workers on how to properly perform nasopharyngeal swabbing in children was largely defective. Of particular concern is the lack of guidelines on the proper depth of swab penetration along the nasal floor in children of different ages.

By using lateral skull radiographs acquired for orthodontic purposes, the present study collected a database of linear measurements of the nostril to nasopharynx distance in a large sample of growing subjects that was stratified by age and gender.

The statistical analysis confirmed that age-related differences in the nostril to nasopharynx distance exist in children, and they should be given due consideration by operators. Since measurements were statistically comparable between the two genders in the great majority of age intervals, male and female data were pooled together for the calculation of descriptive statistics within each age group. The calculated descriptive statistics directed the design of an intuitive, user-friendly swab shaft, helping the operators perform nasopharyngeal swabs in children in a more standardized, controlled, and harmless way (Figure 4). Kaufman et al. warned that performing an effective nasopharyngeal swab without causing significant discomfort to the patient is more complicated than it may appear at first glance and recommended marking out the expected travel distance on the swab until the healthcare worker becomes comfortable with the depth of the nasal cavity [17]. The swab designed in the present study features a shaft with pre-marked age-related signposts that are expected to remarkably increase the reliability and safety of the swabbing procedure.

Concerning the landmarks that were selected for the linear measurements on the lateral skull radiographs, the most antero-superior point of the nostril contour was chosen as an easily traceable point identifying the emergence of the swab shaft from the nose at the skin level. As for the posterior landmark, in the presence of adenoid hypertrophy, the measurement stopped at the adenoid anterior outline. In this regard, it is worth mentioning that whether SARS-CoV-2 can replicate the adenoid tissue remains an unanswered question [5]. It has been reported that SARS-CoV-2 entry factors are highly expressed in nasal epithelial cells, particularly secretory and ciliated cells [21], while lymphocytes do not express them [22]. Additionally, the anatomic barrier of hypertrophic lymphoid tissue overcoming respiratory epithelial viral binding sites has been proposed as a possible explanation for the milder clinical manifestations of COVID-19 disease in children as compared with adults [22]. The issue of the virus replication ability within adenoid tissue is relevant, as it has been reported that in children with adenoid hypertrophy, the test is, in effect, an adenoid swab [5]. The lateral skull radiographs analyzed in the present study confirmed that adenoid hypertrophy most frequently occurs in the 7–9-year age interval (Table 1).

The investigation also pointed out that the nostril to nasopharynx distance stabilized after 11 years of age at around a value of 80 mm (Table 1).

The performed linear measurements of the nostril to nasopharynx distance demonstrated high intra- and inter-examiner repeatability. It was preferred to measure the shortest distance between the nostril and nasopharynx rather than the curvilinear path along the sagittal view of the nasal floor because the latter method revealed lower reliability in a preliminary pilot study, mainly due to the superimposition of the permanent teeth buds with the anatomic structures of interest.

Although the sample was large overall, the 4–4.11 age group had a relatively small size. This may have reflected a low power of the statistical test assessing the between-gender difference. The latter lacked statistical significance, although being quite remarkable. The low numerosity may also have accounted for the relatively large standard deviation and confidence interval that as calculated in this age group and was related to the reduced number of patients undergoing orthodontic treatment at such an early age. Despite the relatively large 95% confidence interval, by referring to its lower limit for the placement of the age-related mark on the shaft, a safety boundary for swab progression was provided, implying that when the mark is aligned with the nostril, the tip is most likely about to reach the nasopharyngeal posterior wall. This observation warns the operator to further advance very cautiously in order to avoid any discomfort or harm to the small child.

As a further development of the research, it is planned to perform the measurements also on radiographs of children belonging to ethnic groups other than the Caucasian race, which was investigated in the present study.

Additionally, a clinical study for the validation of the newly designed nasopharyngeal swab is in progress.

## 5. Conclusions

Based on the nostril to nasopharynx distance measurements taken on lateral skull radiographs, a nasopharyngeal swab prototype was designed, featuring marks along the shaft that pinpoint the proper depth of penetration along the nasal floor at the different ages of childhood and adolescence. The new swab is meant to standardize and ease the nasopharyngeal swabbing procedure for the benefit of healthcare providers, who may have to perform the maneuver on poorly cooperating children or under less-than-ideal sample collection conditions in parking lots, airports, stations, schools, and other ‘point-of-need’ contexts. The newly designed nasopharyngeal swab is valuable for child specimen collection and will still be useful even after the COVID-19 pandemic emergency is overcome since healthcare providers will still resort to nasopharyngeal swabbing as the appropriate test for differential diagnosis of several respiratory infections, which are particularly common in the pediatric age.

## 6. Patents

The authors CG, AVi, and LF are the inventors of several embodiments of the aforementioned swab shaft, currently patent pending.

## Figures and Tables

**Table 1:** Descriptive statistics of the nostril to nasopharynx distance (mm) according to age, gender, and adenoid hypertrophy.

Age Group (Years.Months)	*n*	Mean	SD	95% Confidence Interval	Landmark on Swab Stick
				Statistical Significance of between Gender Difference	
4–4.11	16	56.86	13.41	49.71–64.00	4: At 50 mm distance from the tip
Male	5	65.56	12.81	*p* = 0.054	
Female	11	52.90	12.21		
no adenoid hypertrophy	8	65.51	13.19		
adenoid hypertrophy	8	48.21	6.36		
5–5.11	28	60.75	10.40	56.71–64.78	5: At 57 mm distance from the tip
Male	13	62.03	10.98	*p* = 0.55	
Female	15	59.64	10.13		
no adenoid hypertrophy	19	61.3	11.32		
adenoid hypertrophy	9	59.6	8.65		
6–6.11	32	66.43	10.17	62.76–70.10	6: At 63 mm distance from the tip
Male	13	66.30	8.19	*p* = 0.95	
Female	19	66.52	11.56		
no adenoid hypertrophy	22	67.35	11.51		
adenoid hypertrophy	10	64.41	6.37		
7–7.11	52	71.26	6.07	69.57–72.95	7: At 70 mm distance from the tip
Male	19	70.24	6.42	*p* = 0.36	
Female	33	71.85	5.88		
no adenoid hypertrophy	23	73.82	6.46		
adenoid hypertrophy	29	69.23	4.97		
8–8.11	50	73.63	6.38	71.81–75.44	
Male	28	75.75	5.83	*p* = 0.007	
Female	22	70.93	6.15		
no adenoid hypertrophy	23	76.11	6.05		
adenoid hypertrophy	27	71.52	5.98		
9–9.11	47	76.76	6.65	74.81–78.71	9: At 75 mm distance from the tip
Male	24	78.81	6.17	*p* = 0.029	
Female	23	74.62	6.58		
no adenoid hypertrophy	33	78.60	6.27		
adenoid hypertrophy	14	72.42	5.55		
10–10.11	37	76.69	7.18	74.30–76.69	
Male	21	76.40	6.58	*p* = 0.78	
Female	16	77.08	8.11		
no adenoid hypertrophy	23	78.80	6.78		
adenoid hypertrophy	14	73.23	6.65		
11–11.11	23	83.76	4.50	81.82–85.71	11: At 80 mm distance from the tip
Male	8	85.46	5.76	*p* = 0.195	
Female	15	82.88	3.57		
no adenoid hypertrophy	23	78.80	6.78		
adenoid hypertrophy	0	73.23	6.65		
12–12.11	22	79.45	8.81	75.53–83.36	
Male	11	80.08	10.70	*p* = 0.746	
Female	11	78.81	6.91		
no adenoid hypertrophy	16	82.29	5.75		
adenoid hypertrophy	6	71.86	7.73		
13–14	15	82.95	6.41	79.40–86.50	
Male	9	83.10	7.50	*p* = 0.91	
Female	6	82.73	5.01		
no adenoid hypertrophy	14	83.37	6.44		
adenoid hypertrophy	1	77.1	0		
